# Alternative Ways of Computing the Numerator Relationship Matrix

**DOI:** 10.3389/fgene.2021.655638

**Published:** 2021-07-28

**Authors:** Mohammad Ali Nilforooshan, Dorian Garrick, Bevin Harris

**Affiliations:** ^1^Livestock Improvement Corporation, Hamilton, New Zealand; ^2^AL Rae Centre for Genetics and Breeding, School of Agriculture, Massey University, Palmerston North, New Zealand

**Keywords:** pedigree, numeric relationship matrix, inverse, Cholesky decomposition, conjugate gradient, inbreeding coefficient, parallel computing

## Abstract

Pedigree relationships between every pair of individuals forms the elements of the additive genetic relationship matrix (**A**). Calculation of **A**^−1^ does not require forming and inverting **A**, and it is faster and easier than the calculation of **A**. Although **A**^−1^ is used in best linear unbiased prediction of genetic merit, **A** is used in population studies and post-evaluation procedures, such as breeding programs and controlling the rate of inbreeding. Three pedigrees with 20,000 animals (20K) and different (1, 2, 4) litter sizes, and a pedigree with 180,000 animals (180K) and litter size 2 were simulated. Aiming to reduce the computation time for calculating **A**, new methods [Array-Tabular method, (^**T**^−1^)−1^ instead of **T** in Thompson's method, iterative updating of **D** in Thompson's method, and iteration by generation] were developed and compared with some existing methods. The methods were coded in the R programming language to demonstrate the algorithms, aiming for minimizing the computational time. Among 20K, computational time decreased with increasing litter size for most of the methods. Methods deriving **A** from **A**^−1^ were relatively slow. The other methods were either using only pedigree information or both the pedigree and inbreeding coefficients. Calculating inbreeding coefficients was extremely fast (<0.2 s for 180K). Parallel computing (15 cores) was adopted for methods that were based on solving **A**^−1^ for columns of **A**, as those methods allowed implicit parallelism. Optimizing the code for one of the earliest methods enabled **A** to be built in 13 s (faster than the 31 s for calculating **A**^−1^) for 20K and 17 min 3 s for 180K. Memory is a bottleneck for large pedigrees but attempts to reduce the memory usage increased the computational time. To reduce disk space usage, memory usage, and computational time, relationship coefficients of old animals in the pedigree can be archived and relationship coefficients for parents of the next generation can be saved in an external file for successive updates to the pedigree and the **A** matrix.

## 1. Introduction

Co-ancestry or kinship is a fundamental characteristic in population and quantitative genetics. Co-ancestry is reflected by the coefficient of relationship, which is defined as the probability that two individuals share identical alleles by descent (Falconer and Mackay, 1996). The additive genetic relationship between two individuals is twice their co-ancestry. Wright (1922) computed relationships based on path coefficients (sum over all the paths of the products of the coefficients in a path that connects two individuals through common ancestors). Working with large pedigrees, it is hard to keep track of all the paths and computationally efficient methods are required. Additive genetic relationships between individuals in a pedigree form the elements of the additive genetic relationship matrix, also called the numerator relationship matrix (**A**) and are the basis of the variance–covariance of breeding values for animals in the pedigree.

Since the development of best linear unbiased prediction (BLUP) methodology (Henderson et al., 1959) up to recent advanced methods for genomic evaluation (Fernando et al., 2016), the **A**^−1^ matrix has been a critical element used in computations for predicting genetic merit of individuals. It allows algorithms to exploit information on relatives for predicting genetic merit accounting for the genetic (co)variance structure among individuals in the population. More details about BLUP is given by Henderson (1975a), where it is shown how unbiasedness and minimum prediction error of predictors are met in populations under selection. Henderson (1976) and Quaas (1976) presented methods of directly deriving **A**^−1^ from the pedigree without the need to form **A**. Matrix **A**^−1^ has rows and columns corresponding to animals in the pedigree and it is very sparse. Many studies have been performed to improve the computational efficiency in constructing **A**^−1^ and in particular the diagonal elements of **A**, which are required for the construction of **A**^−1^ (Tier, 1990; Meuwissen and Luo, 1992; Colleau, 2002; Sargolzaei and Iwaisaki, 2005; Sargolzaei et al., 2005). However, there has been relatively little attention to the efficient calculation of off-diagonal elements of **A**. This is particularly because there is less demand for elements of **A** compared to elements of **A**^−1^. Also, the calculation of **A** is typically computationally more demanding. **A**^−1^ is sparser than **A** and calculations for each individual only rely on the individual and its parents. In **A**, the relationship coefficient between individuals *i* and *j* equals to the average of the relationship coefficients between the parents of *j* with *i* (0 for unknown parents). Although **A** is not needed for routine genetic evaluations, there is need for numerical values of some or all of its elements in population and gene flow studies, conservation programs, controlling the rate of inbreeding and in designing breeding programs. The aim of this study is to review existing methods and introduce new algorithms for calculating **A**. Runtime comparisons of the approaches are provided.

## 2. Materials

Three pedigree structures (PED1, PED2, and PED3) with litter sizes of 1, 2, or 4 were simulated and tested. The pedigree simulation was performed using the R package “pedSimulate” (Nilforooshan, 2021) following the steps below:

1. Starting with 200 base population individuals, representing an equal number of foundation males and females.
2. Those foundation individuals were randomly mated (no selection or pre-mating mortality) to produce the next generation.
3. Pre-mating mortality rate was 3% in subsequent generations.
4. There was no overlap of generations for females, but there was one generation overlap for males.
5. Note that 80% of mature females and 20% of mature males were selected as parents of the next generation, then randomly mated.
6. Data were simulated generation after generation until reaching a population size of 20,000 individuals.
7. After the pedigree was simulated, 10% of dams and 20% of sires were randomly set to missing in non-base generations.

Another pedigree structure (PED4) was simulated similar to PED2, but with a population size of 180,000 individuals. PED4 was specifically created to be tested on the fastest methods based on the results from the three other pedigrees, and to find out about computational limitations and hardware requirements for a larger pedigree. The pedigree files are provided in the data repository (Data Availability Statement).

R statistical software (R Core Team, 2019) was used throughout this study. The R package “pedigreemm” (Vazquez et al., 2010) was used for calculating inbreeding coefficients and the **A**^−1^ matrix, and R packages “doParallel” (Wallig et al., 2020b) and “foreach” (Wallig et al., 2020a) were used for parallel processing, when the main task could be split into independent threads. Libraries LAPACK and SuiteSparse were exploited by using the R package “Matrix” (Bates and Maechler, 2019) for operations on sparse matrices. Data, Shell scripts, R code, and R functions used in this study are available in the data repository (Data Availability Statement).

Computations and benchmarking were performed on an Ubuntu server 20.04 LTS Amazon machine image. A few types of cloud machines from the R5, C5, and T2 families of the Amazon elastic compute cloud were used for the computations. Specifications of the cloud machine families are given in the Appendix, and specifications of each machine (number of virtual CPUs and read-only memory (RAM) capacity) are given in section 5.

## 3. Methods

### 3.1. Tabular Method

The history of the tabular method goes back to early works done by Emik and Terrill (1949) and Cruden (1949). This method is based on simple rules. First, individuals are ordered in the pedigree to ensure parents appear before their progeny. Considering *s* and *d* being sire and dam of *j*, and individual *i* appeared in the pedigree before *j*, *A*_*ij*_ = (*A*_*si*_+*A*_*di*_)/2. If a parent (e.g., *s*) is unknown, *A*_*si*_ = 0. If both parents *s* and *d* are known, *A*_*jj*_ = 1+*F*_*j*_ = 1+*A*_*sd*_/2. The original method loops over rows, and columns within a row, to sequentially calculate matrix elements. Because **A** is symmetric, *n*(*n*+1)/2 of *n*^2^ elements need to be calculated. This method is coded in function buildA of R package “ggroups” (Nilforooshan and Saavedra-Jiménez, 2020). Using array programming techniques (vectorized calculations), we optimized this method (Array-Tabular or method 3.1.2) to loop only over columns, which is expected to speed up the calculations. The *j*th array is calculated from the *s*th (for known *s*) and the *d*th array (for known *d*). This method is coded in R function tabularA (Data Availability Statement), which requires a pedigree data frame as input.

### 3.2. Thompson's Method

Applying the LDL factorization, **A** = **TDT**′, where **T** is a lower triangle matrix, and **D** is a diagonal matrix. In this method (Thompson, 1977), **T** and **D** matrices required to calculate **A** are derived indirectly (*i.e*., no direct factorization or decomposition). For individual *j*, off-diagonal elements that were initialized with 0 are changed to *T*_*ij*_ = (*T*_*si*_+*T*_*di*_)/2 if both parents *s* and *d* are known, and to *T*_*ij*_ = *T*_*si*_/2 if one parent (e.g., *s*) is known. The diagonal elements of **D** that were initiated with 1 change to *D*_*jj*_ = (2−*F*_*s*_−*F*_*d*_)/4 if both parents are known, and to *D*_*jj*_ = (3−*F*_*s*_)/4 if one parent (e.g., *s*) is known. This method is coded in R function TDTp (Data Availability Statement). This function requires the pedigree data frame and a corresponding vector of inbreeding coefficients as input.

Alternatively, (**T**^−1^)^−1^ may be used instead of **T** (method 3.2.2), where **T**^−1^ = **I**−**J** and **J** is the lower triangle parent incidence matrix, with coefficients 0 and 0.5. R function TDTp2 (Data Availability Statement) calculates **TDT**′ via **T^−1^**.

### 3.3. Iterative Updating of D in Thompson's Method

Using Thompson's Method, **A** = **TDT**′. The initial **D** (**D**_0_) is calculated ignoring inbreeding coefficients in the population (*D*_*jj*_ = 1−*p*_*j*_/4, where *p*_*j*_ is the number of known parents for individual *j*). Using sire and dam design matrices (**J**_*s*_ and **J**_*d*_) with coefficients 0 and 0.5, matrix **D** and consequently **A** are updated in each iteration until diag(**A**) remains unchanged.

In iteration *i*, ${\text{A}}_{i}={\text{T}\text{D}}_{i}{\text{T}}^{\prime }$, diag(**A**_*i*+1_) and **D**_*i*+1_ are calculated. Note that diag(**A**_*i*+1_) is calculated prior to calculating off-diagonal elements of **A**_*i*+1_, where $\text{diag}\left({\text{A}}_{i+1}\right)=1+2\times \text{diag}\left({\text{J}}_{s}{\text{A}}_{i}{\text{J}}_{d}^{\prime }\right)$. For individual *j* with sire *s* and dam *d* that meets the three conditions of (1) *A*_*j*_*j*__*i*__>diag(_**A**_*i*+1_)*j*_, (2) *A*_*s*_*s*__*i*__ = diag(_**A**_*i*+1_)*s*_ and (3) *A*_*d*_*d*__*i*__ = diag(_**A**_*i*+1_)*d*_, *D*_*j*_*j*__(*i*+1)__ is calculated as 1−(*A*_*s*_*s*__*i*__+*A*_*d*_*d*__*i*__)/4. If a parent (e.g., *s*) is unknown, then *A*_*s*_*s*__*i*__ = 0. The iterations continue until there is no individual *j* left with *A*_*j*_*j*__*i*__>diag(_**A**_*i*+1_)*j*_+ϵ, where ϵ is a small positive (tolerance) value, which was set to 1e–5 in this study. This method is coded in R function AiterD (Data Availability Statement), and it requires the pedigree data frame and the tolerance value as input.

### 3.4. Henderson's Method

Using the concepts of Cholesky decomposition, **A** can be written as **A** = **LL**′, where **L** is a lower triangle matrix with non-zero diagonal elements. Henderson (1975b) introduced a method for deriving **L** indirectly to calculate **A**. If the definition of *s* and *d* change from sire and dam of *j* to parents of *j*, where *s*<*d*, calculation of **L** can be formulated as:

1. **L** = **I**
2. If only one parent (e.g., *s*) is known:
  a. *L*_*ij*|*i* ≤ *s*_ = *L*_*si*_/2
  b. $L_{jj}=(1-\overset{s}{\underset{i=1}{\sum }}L_{ij}^{2}{)}^{\frac{1}{2}}$
3. If both parents are known:
  a. *L*_*ij*|*i* ≤ *s*_ = (*L*_*si*_+*L*_*di*_)/2
  b. *L*_*ij*|*s*<*i* ≤ *d*_ = *L*_*di*_/2
  c. $L_{jj}=(1+\frac{1}{2}\overset{s}{\underset{i=1}{\sum }}L_{si}L_{di}-\overset{s}{\underset{i=1}{\sum }}L_{ij}^{2}{)}^{\frac{1}{2}}$

Calculation of **L**′ is coded in R function getLp (Data Availability Statement), and it requires a pedigree data frame as input.

### 3.5. Iteration by Generation

The previous methods iterate over individuals with at least one known parent. Number of loops can increase the computational time. Thus, a method that minimizes loops (e.g., generations rather than individuals) could be beneficial. In this method, the **A** matrix is successively enlarged and completed generation by generation. The base (0) generation, irrespective of the date of birth, includes individuals with both parents unknown, and **A**_0_ = **I**. Individuals in generation *i* (*i* > 0) have a known parent in generation *i* – 1 and the other parent either unknown or in generation < *i*. For generation *i*,

$$\begin{array}{l} {\text{A}}_{i}=\left[\begin{array}{c} \text{I}\\ {\text{J}}_{i} \end{array}\right]{\text{A}}_{i-1}{\left[\begin{array}{c} \text{I}\\ {\text{J}}_{i} \end{array}\right]}^{\prime }+\left[\begin{array}{cc} 0 & 0\\ 0 & \text{diag}\left({\text{o}}_{i}\right) \end{array}\right]\\ =\left[\begin{array}{cc} {\text{A}}_{i-1} & {\text{A}}_{i-1}{\text{J}}_{i}^{\prime }\\ {\text{J}}_{i}{\text{A}}_{i-1} & {\text{J}}_{i}{\text{A}}_{i-1}{\text{J}}_{i}^{\prime }+\text{diag}\left({\text{o}}_{i}\right) \end{array}\right], \end{array}$$

where ${\text{o}}_{i}=1+2\times \text{diag}\left({\text{J}}_{s_{i}}{\text{A}}_{i-1}{\text{J}}_{d_{i}}^{\prime }\right)-\text{diag}\left({\text{J}}_{i}{\text{A}}_{i-1}{\text{J}}_{i}^{\prime }\right)$, **J**_*i*_ is the parent incidence matrix with elements of 0.5 and 0, rows corresponding to individuals in generation *i* and columns corresponding to individuals in generations < *i*. **J**_*s*_*i*__ and **J**_*d*_*i*__ are sire and dam incidence matrices with elements of 0.5 and 0, and **J**_*i*_ = **J**_*s*_*i*__+**J**_*d*_*i*__. If the definition of *s* and *d* change from sire and dam to parents, where *s*<*d*, **A**_*i*−1_ can be replaced with its upper triangle without diagonal elements.

For example, consider the following pedigree with columns corresponding to individual, sire and dam ID:

$$\begin{array}{ccc} 1 & 0 & 0\\ 2 & 0 & 0\\ 3 & 1 & 0\\ 4 & 1 & 2\\ 5 & 3 & 4\\ 6 & 1 & 4\\ 7 & 5 & 6 \end{array}$$

Individuals 1 and 2 are assigned to the base generation, individuals 3 and 4 to generation 1, individuals 5 and 6 to generation 2, and individual 7 to generation 3. The matrices involved in the calculation of **A** are presented below:

$$\begin{array}{l} \begin{array}{cc} {\text{A}}_{0} & ={\text{I}}_{2},\\ {\text{J}}_{s_{1}} & =\left[\begin{array}{cc} 0.5 & 0\\ 0.5 & 0 \end{array}\right],\;{\text{J}}_{d_{1}}=\left[\begin{array}{cc} 0 & 0\\ 0 & 0.5 \end{array}\right],\\ {\text{J}}_{s_{2}} & =\left[\begin{array}{cccc} 0 & 0 & 0.5 & 0\\ 0.5 & 0 & 0 & 0 \end{array}\right],\;{\text{J}}_{d_{2}}=\left[\begin{array}{cccc} 0 & 0 & 0 & 0.5\\ 0 & 0 & 0 & 0.5 \end{array}\right],\\ {\text{J}}_{s_{3}} & =\left[\begin{array}{cccccc} 0 & 0 & 0 & 0 & 0.5 & 0 \end{array}\right],\;{\text{J}}_{d_{3}}=\left[\begin{array}{cccccc} 0 & 0 & 0 & 0 & 0 & 0.5 \end{array}\right]. \end{array} \end{array}$$

This method is coded in R function iterGen (Data Availability Statement), and it requires a pedigree data frame as input. This function is written in a way that there is no need to form **J**_*s*_*i*__, **J**_*d*_*i*__ or **J**_*i*_, nor to do any matrix multiplications.

### 3.6. Direct Solving of **A**^−1^

Calculation of **A**^−1^ is typically computationally less extensive than the calculation of **A**, and there are fast algorithms for computing inbreeding coefficients (Tier, 1990; Meuwissen and Luo, 1992; Colleau, 2002; Sargolzaei and Iwaisaki, 2005; Sargolzaei et al., 2005), which are required for the derivation of **A**^−1^. One way of obtaining **A** is via solving equations involving **A**^−1^. In this approach, **A**^−1^ was directly solved to form **A**, using the solve function in R.

### 3.7. Solving **A**^−1^ for Columns of A

Evidently, **A**^−1^**A** = **I**. This method is based on solving the equation system **A**^−1^**x** = **b**, where **x** is the *i*th column of **A** and **b** is the vector of **0** with the *i*th element (corresponding to the *i*th column of **A**) set to 1. This method is coded in R function solveAi (Data Availability Statement), and it requires matrix **A**^−1^ as input.

### 3.8. Iterative Solving **M**^−1^**A**^−1^ for Columns of A

This method is similar to Solving **A**^−1^ for Columns of **A**, but the pre-conditioned conjugate gradient (PCG) algorithm is used to solve the equation system. In PCG, a pre-conditioner matrix (**M**^−1^) is used for conditioning the linear equation system, and it exploits the symmetric positive definite structure of the coefficient matrix. The PCG algorithm is coded in R function pcg. Function pcgAi is a wrapper of function pcg over the columns of **A**. Both functions are available in the data repository (Data Availability Statement). For each column of **A**, the equation system **M**^−1^**A**^−1^**x** = **M**^−1^**b** is iteratively solved, where **x** is the *i*th column of **A**. Vector **b** (as defined in Solving **A**^−1^ for Columns of **A**) was used as prior for **x**. One may use previously solved columns of **A** as prior for the current **x** by copying the previously solved columns of **A** to the corresponding rows. However, it creates dependency between solving each column of **A** with its previous columns. In order to take advantage of parallel processing, we chose not to use this option. The PCG convergence criterion was set to 1e–5. We assumed that the resulting **A** matrix is symmetric. If not, there would be additional computational cost for making it symmetric [i.e., **A** = (**A**+**A**′)/2]. Matrices **A**^−1^ and **M**^−1^ are input to function pcgAi. If matrix **M**^−1^ is not defined with the argument Minv, or Minv = NULL, the conjugate gradient method is used instead of PCG, which makes no use of a pre-conditioner matrix. A few alternatives were tested as **M**^−1^:

#### 3.8.1. **M**^−1^ = diag(1/diag(**A**^−1^))

#### 3.8.2. **M**^−1^ = **D**

Matrix **D** is as defined in Thompson's Method. R function getD (Data Availability Statement) calculates diag(**D**) from the pedigree data frame and the corresponding vector of inbreeding coefficients.

#### 3.8.3. ${\text{M}}^{-1}={\text{D}}_{0}$

Matrix **D**_0_ is as defined in Iterative Updating of **D** in Thompson's Method. R function getD0 (Data Availability Statement) calculates diag(**D**_0_) from the pedigree data frame.

## 4. Method Features

### 4.1. Tabular Method

The tabular method is straightforward based on very simple rules. The *cons* of this method are as follows: (1) It loops over rows of **A** and over columns 1 to *i* in row *i*. Loops can be skipped for animals with both parents unknown. There is an additional cost of calculating an inbreeding coefficient in every loop on a row, if both parents of the animal are known. (2) Due to dependencies (progeny on parents) between calculations, computations cannot easily be parallelized. (3) Because the method involves updating **A** in every loop, the method could not take advantage of libraries for sparse matrices. Such libraries reduce memory usage and computational complexity of matrix operations.

We used R package “Matrix” (Bates and Maechler, 2019) for sparse matrices. However, iteratively forming or updating elements of a matrix initially built in a sparse format slowed down the procedure considerably. We think a possible reason was that every time the matrix was getting updated the memory had to be re-allocated. Therefore, we used R package “Matrix” (equipped with libraries LAPACK and SuiteSparse) for whole matrix operations rather than for updating elements of the matrix or appending rows/columns to it.

The benefit of the Array- Tabular Method over the Tabular Method was reducing the number of loops with replacing inner (on columns of **A**) loops with vectorized operations. Here, we chose to loop over columns than rows, because operations on columns are faster than operation on rows in modern programming languages such as R and Julia.

### 4.2. Thompson's Method

Matrix **T** and vector **d** (**d** = diag(**D**)) had to be created iteratively with an iteration over every animal with a known parent. As a result, the libraries for sparse matrices (LAPACK and SuiteSparse) were not used at this (updating) stage. The cost of making **T** and **d** was slightly lower than the cost of directly making **A** via the Tabular Method. The main benefit of this method was that after creating **T** and **d**, it was possible to take advantage of libraries for sparse matrices. Matrix **T** was converted to a sparse format and vector **d** was converted to the diagonal sparsely formatted **D**. Similar to the Array- Tabular Method, inner loops were replaced by vectorized operations for making **T**. There were additional costs involved with storing **T** and **D** in the memory and matrix multiplications (**TDT**′), however those were considerably reduced by making use of the libraries for sparse matrices (LAPACK and SuiteSparse).

We tested (**T**^−1^)^−1^ instead of **T** and the reason was that **T**^−1^ is much sparser than **T** (which has off-diagonal elements of −0.5 in each row for each known parent of the individual and a diagonal element of 1), but it can be built immediately, because there are no dependencies between rows of **T**^−1^. On the other hand, there is a computational cost involved for inverting the lower triangle sparse matrix **T**^−1^.

### 4.3. Iterative Updating of D in Thompson's Method

The aim of this method was to remove the dependency on inbreeding coefficients that existed in Thompson's Method. The one-off computational cost for the calculation of **D** is replaced with an iterative procedure for updating **D**_*i*_ in iteration *i*, starting with **D**_0_ with 3 possible values depending on the number of known parents of the individual. There are matrix multiplication costs (${\text{T}\text{D}}_{i}{\text{T}}^{\prime }$) involved in each iteration. The number of iterations to update and stabilize **D**_*i*_ is much lower than the number of iterations to form **D** from inbreeding coefficients. However, given the cost of updating **D**_*i*_, this method might only be justifiable over Thompson's Method if the inbreeding coefficients are not available or the number of iterations to update **D**_*i*_ is very low. Using (**T**^−1^)^−1^ rather than **T**, this method does not require ordering animals in the pedigree.

### 4.4. Henderson's Method

Compared to Thompson's Method, this method requires less matrix multiplications (**LL**′ vs. **TDT**′). However, calculating **L** is computationally more demanding than calculating **T**. Matrices **L** and **T** have the same sparsity. Matrix **L** is created iteratively, and then sparsely formatted to reduce the matrix multiplication cost using the libraries for sparse matrices, provided by R package “Matrix.”

### 4.5. Iteration by Generation

This method is independent from inbreeding coefficients in the population. Most of the previous methods iterate over rows of **A** and even columns 1 to *i* within row *i* (e.g., Tabular Method). This method on the other hand iterates over generations. In each generation *i*, a parent incidence matrix (**J**_*i*_) needs to be created and there are matrix multiplications and summations involved. However, in the code, these matrix operations were omitted by detecting parents of the current generation and looping over parents within the generation. As a result, the number of loop iterations are reduced considerably compared to the methods looping over individuals, especially if there are many progeny per parent per generation. Calculations for progeny per parent per generation were done using vectorized operations. Due to matrix updating, libraries for sparse matrices were not used.

### 4.6. Direct Solving of **A**^−1^

Calculation of **A**^−1^ is typically very fast and computationally less demanding (operations and memory usage) than the calculation of **A**. Solving **A** from **A**^−1^ does not involve matrix updating and **A**^−1^ is very sparse. Therefore, there is great opportunity in using libraries for sparse matrices. Another advantage in using **A**^−1^ to derive **A** is the lack of calculation dependencies. As a result, the solving procedure can be done at once and in parallel. The first method of solving **A** from **A**^−1^ is of course direct solving of **A**^−1^. Explicit parallelism was an option. However, in this study we limited the use of parallel processing to implicit parallelism.

### 4.7. Solving **A**^−1^ for Columns of A

Quantitative fields of science deal with solving systems of linear equations. Equation systems can be presented in a matrix form, where the coefficient matrix of predictions (here, **A**^−1^) multiplied by the vector of predictions (here, the *i*th column of **A**) equals to the right-hand-side vector (here, a vector of zeros with the *i*th element equal to 1). The vector of predictions is to be predicted by solving the equation system. Solving for columns of **A** was parallelized implicitly. Though, solving each column of **A** could be parallelized explicitly, it had no justification over explicit parallelism of Direct Solving of **A**^−1^. For this method, we took advantage of the libraries for sparse matrices via R package “Matrix.”

### 4.8. Iterative Solving **M**^−1^**A**^−1^ for Columns of A

The iterative PCG algorithm is commonly used in quantitative genetics to solve mixed model equations. It is preferable for solving large and sparse equation systems, where direct solving of the coefficient matrix is not feasible. Pre-conditioner matrices diag(1/diag(**A**^−1^)), **D** and **D**_0_ were tested. This method took advantage of implicit parallel processing for independently solving columns of **A** and libraries for sparse matrices.

## 5. Results

### 5.1. Simulated Pedigrees

Table 1 provides information about the simulated pedigrees. With a constant pedigree size (PED1, PED2, and PED3), the number of generations and the total number of parents decreased with increasing litter size. The number of inbred individuals decreased with increasing litter size (Table 1) since fewer generations were represented. However, the average inbreeding coefficients among inbred individuals increased with litter size and were 0.008, 0.013, and 0.030 for PED1, PED2, and PED3, respectively. The maximum inbreeding coefficient was 0.25 for all the pedigrees.

**Table 1:** Description of the simulated pedigrees.

**Number of**	**PED1**	**PED2**	**PED3**	**PED4**
Individuals	20,000	20,000	20,000	180,000
Litter size	1	2	4	2
Generations	15	9	6	12
Sires	3,507	2,084	1,449	21,985
Dams	6,271	5,012	4,176	51,890
Missing sires	4,160	4,160	4,160	36,160
Missing dams	2,180	2,180	2,180	18,180
Missing both parents	582	595	630	3,808
Inbred individuals	3,240	2,025	930	62,449

### 5.2. Pedigrees With 20,000 Animals

Table 2 shows elapsed time for the calculation of **A**, using different methods for PED1, PED2, and PED3. The elapsed times presented in Table 2 were recorded on an “r5.large” cloud machine with 2 cores, and 16 Gb of RAM for the methods that were making no use of parallel processing, and a “c5.4xlarge” cloud machine with 16 cores and 32 Gb of RAM for the methods that were making use of parallel processing (3.7 and 3.8). The new methods (3.1.2, 3.2.2, 3.3, and 3.5) were among the 7 (of 12) fastest methods. The five fastest methods were 3.1.2 being the fastest to 3.4, 3.2.1, 3.2.2, and 3.5. Except for the methods 3.8.1, 3.8.2, and 3.8.3, the runtime decreased from PED1 to PED3, as a result of a greater litter size, less number of generations and less number of parents in the same population size.

**Table 2:** Elapsed time (MM:SS) for the calculation of **A** matrix using different methods for different pedigree.

**Method**	**PED1**	**PED2**	**PED3**
3.1.1: Tabular Method	01:39	01:37	01:36
3.1.2: Array- Tabular Method	00:13	00:13	00:13
3.2.1: Thompson's Method	00:47	00:41	00:34
3.2.2: (**T**^−1^)^−1^ instead of **T** in Thompson's Method	01:03	00:58	00:51
3.3 : Iterative Updating of **D** in Thompson's Method	04:11	02:05	01:01
3.4 : Henderson's Method	00:42	00:37	00:32
3.5 : Iteration by Generation	02:13	01:13	00:45
3.6 : Direct Solving of **A**^−1^	04:08	02:32	01:43
3.7 : Solving **A**^−1^ for Columns of **A**^p^	07:31	05:53	05:35
3.8 : Iterative Solving **M**^−1^**A**^−1^ for Columns of **A**^p^	
3.8.1 : **M**^−1^ = diag(1/diag(**A**^−1^))	11:42	12:10	11:48
3.8.2 : **M**^−1^ = **D**	15:13	16:42	18:45
3.8.3 : ${\text{M}}^{-1}={\text{D}}_{0}$	15:25	17:08	18:53

*p, parallel processing using 15 computational cores; PED1, The pedigree of 20,000 individuals and litter size of 1; PED2, The pedigree of 20,000 individuals and litter size of 2; PED3, The pedigree of 20,000 individuals and litter size of 4; **M**^−1^ is the pre-conditioner matrix for the pre-conditioned conjugate gradient algorithm; ***D***is a diagonal matrix introduced in Thompson's Method; **D**_0_ is the starting ***D***in Iterative Updating of **D** in Thompson's Method*.

Compared to Thompson's Method, Iterative Updating of **D** in Thompson's Method was independent from inbreeding coefficients being known in the population. However, using fast algorithms for calculating inbreeding coefficients (Tier, 1990; Meuwissen and Luo, 1992; Colleau, 2002; Sargolzaei and Iwaisaki, 2005; Sargolzaei et al., 2005), these coefficients can be computed in a short time for large populations. Calculation of inbreeding coefficients took less than 0.01 s for PED1, PED2, and PED3, shorter with less number of generations (larger litter sizes). Consequently, calculating **D** based on known inbreeding coefficients is faster than starting with **D**_0_ with no knowledge on inbreeding coefficients in the population and then iteratively converging to **D**.

Calculation of **A**^−1^ was fast and efficient. It took 31.4, 31.3, and 31.2 s for PED1, PED2, and PED3 to calculate **A**^−1^. Unlike **A**, **A**^−1^ is sparse and calculations for each individual involves the individual and its parents rather than information on all relatives. Consequently, a possibility for the calculation of **A** is via **A**^−1^. Off-diagonal elements of **A**^−1^ showed 99.98% sparsity for all the pedigrees. On the other hand, off-diagonal elements of **A** showed 58.04%, 70.51%, and 85.14% sparsity for PED1, PED2, and PED3, respectively. The average of non-zero off-diagonal elements of **A** was 0.007, 0.011, and 0.023, and the maximum relatedness was 0.750, 0.750, and 0.812 for PED1, PED2, and PED3, respectively. Direct Solving of **A**^−1^ was faster than Solving **A**^−1^ for Columns of **A**, and Solving **A**^−1^ for Columns of **A** was faster than Iterative Solving **M**^−1^**A**^−1^ for Columns of **A**.

We made use of the parallel version of LAPACK, ScaLAPACK library for high-performance linear algebra routines for parallel distributed memory machines to do Solving **A**^−1^ for Columns of **A** on 15 cores of an “r5.4xlarge” machine with 16 cores and 128 Gb of RAM. However, the procedure was crushed due to the high memory usage.

Three diagonal pre-conditioner (**M**^−1^) matrices were tested for Iterative Solving **M**^−1^**A**^−1^ for Columns of **A**. We did not find any notable difference between **D** and **D**_0_ used as **M**^−1^ in time to calculate **A**. On the other hand, diag(1/diag(**A**^−1^)) was a better **M**^−1^ and it further reduced the elapsed time considerably (Table 2). The differences between the elapsed time of the PCG-based methods were mainly due to the convergence of the PCG algorithm rather than the elapsed time to form **M**^−1^. Forming diag(1/diag(**A**^−1^)) and **D**_0_ each took 0.02 s, and it took 1 s to form **D** for PED1, PED2, and PED3. Block pre-conditioner matrices **TT**′ and ${\text{T}\text{D}}_{0}{\text{T}}^{\prime }$ were also tested (results not shown). Besides being computationally more expensive to build, those matrices considerably increased the computational time likely due to increased matrix multiplication costs per PCG iteration per column of **A**.

Root of mean squared error (RMSE, excluding the upper diagonal elements of **A**) for the methods of Solving **A**^−1^ for Columns of **A** and Iterative Solving **M**^−1^**A**^−1^ for Columns of **A** are presented in Table 3. Even with the low strict PCG convergence criterion of 1e–5, RMSE values were very close to zero for most of the methods. The method of Solving **A**^−1^ for Columns of **A** produced considerably lower RMSE values than the method of Iterative Solving **M**^−1^**A**^−1^ for Columns of **A**. There was no difference between the PCG methods using **D** and **D**_0_ as **M**^−1^. Using diag(1/diag(**A**^−1^)) as **M**^−1^ produced the highest RMSE values.

**Table 3:** Root of mean squared error for the lower triangle elements of **A** for the methods of solving **A**^−1^ and **M**^−1^**A**^−1^ for columns of **A**, where **M**^−1^ is the pre-conditioner matrix for the pre-conditioned conjugate gradient algorithm.

**Method**	**PED1**	**PED2**	**PED3**
3.7 : Solving **A**^−1^ for Columns of **A**	4e–17	5e–17	4e–17
3.8 : Iterative Solving **M**^−1^**A**^−1^ for Columns of **A**	
3.8.1 : **M**^−1^ = diag(1/diag(**A**^−1^))	1.2e–4	1.5e–4	1.7e–4
3.8.2 : **M**^−1^ = **D**	7.5e–5	9e–5	1.2e–4
3.8.3 : ${\text{M}}^{-1}={\text{D}}_{0}$	7.5e–5	9e–5	1.2e–4

*PED1, The pedigree of 20,000 individuals and litter size of 1; PED2, The pedigree of 20,000 individuals and litter size of 2; PED3, The pedigree of 20,000 individuals and litter size of 4; **D** is a diagonal matrix introduced in Thompson's Method; **D**_0_ is the starting **D** in Iterative Updating of **D** in Thompson's Method *.

Wherever possible, libraries for sparse matrices should be used to reduce the memory usage and computational complexity for sparse matrices. Table 4 shows the memory usage by matrices **A**, **A**^−1^, **T**, **T**^−1^, **L**, and **D** in sparse format. Those 20,000 ×20,000 matrices occupied more than 32e+8 bytes of memory when not stored in sparse format.

**Table 4:** Memory usage (bytes) of matrix **A** and other matrices used to calculate **A**, when stored in sparse format, using R package “Matrix.”

**Matrix**	**PED1**	**PED2**	**PED3**
**A**	1,007,355,312	708,069,624	356,891,384
**A** ^−1^	3,456,376	3,397,608	3,361,008
**T**	6,680,136	4,618,272	2,995,416
**T** ^−1^	725,424	725,424	725,424
**L**	6,680,136	4,618,272	2,995,416
**D**	161,240	161,240	161,240

*PED1, The pedigree of 20,000 individuals and litter size of 1; PED2, The pedigree of 20,000 individuals and litter size of 2; PED3, The pedigree of 20,000 individuals and litter size of 4; **T** is a lower triangle matrix introduced in Thompson's Method ; **D** is a diagonal matrix introduced in Thompson's Method ; **D**_0_ is the starting **D** in Iterative Updating of **D** in Thompson's Method *.

### 5.3. Pedigree With 180,000 Animals

The Array- Tabular Method and the non-optimized Tabular Method (loops over rows and columns within each row) were tested on a larger pedigree with 180,000 animals (PED4), and it took them 17 min 3 s and 2 h 20 min 34 s time to calculate **A**, respectively. Calculating **A** for such a pedigree required a much larger memory than for a pedigree with 20,000 animals. Therefore, an “r5.8xlarge” cloud machine was used, which has 32 cores and 256 Gb of RAM. Inbreeding coefficients were calculated in 0.13 s. Other methods were not tested for PED4, either because those performed slow for the pedigrees with 20,000 animals or they failed due to the shortage of memory. Calculation of **T**^−1^, **T**′, **L**′, **D**, and **D**_0_ took 2 min 16 s, 4 min 8 s, 5 min 2 s, 9.4 s, and 0.01 s, respectively. However, there was a shortage of memory for matrix multiplications to derive **A**. Also, the calculation of **A**^−1^ (using function getInv from R package “pedigreemm”) failed due to the shortage of memory.

## 6. Discussion

Calculating matrix **A** is typically computationally more demanding than the calculation of **A**^−1^, because except for individuals missing both parents, all the coefficients are to be calculated. Various methods of calculating **A**, from pedigree or from **A**^−1^, with/without knowledge on inbreeding coefficients, and with/without the possibility of implicit (independent) parallelism were tested and developed. Availability of information on inbreeding coefficients was not an issue, as inbreeding coefficients for large populations can be calculated in a very short period of time. Although the calculation of **A**^−1^ is straightforward and fast, the methods based on solving **A** from **A**^−1^ were relatively slow. These methods are widely and commonly used for solving linear equation systems, such as generalized linear models and linear mixed models. However, unlike **A** and **A**^−1^, coefficient matrix (left-hand-side matrix) of those models is complex and does not have any known properties, except that it is symmetric and it should be positive definite. Making use of the known properties of **A**, it can be calculated directly from pedigree with no additional information needed (inbreeding coefficients are required for Thompson's Method).

Direct Solving of **A**^−1^ as a big single task was faster than Solving **A**^−1^ for Columns of **A** with 20,000 smaller tasks (for pedigrees with 20,000 individuals), despite those were distributed over 15 computational cores rather than a single core. Further increasing the number of computational cores for the methods using parallel processing reduces their computational time, but the price per computational unit increases. Presumably, the sparsity of **A**^−1^ justified Direct Solving of **A**^−1^ over Solving **A**^−1^ for Columns of **A**. In the case of this study, because the treads of split task were independent from each other, parallelism overhead was minimized. Increased parallelism overhead minimizes the benefit from increased number of computational cores. Depending on the size and sparsity of the matrix, there are situations where direct inversion is preferred over distributed (e.g., Solving **A**^−1^ for Columns of **A**) and iterative procedures.

### 6.1. Other Ways of Solving A From **A**^−1^

There are other ways of solving **A** from **A**^−1^, which were not covered in this study. Examples are the conjugate gradient algorithm (i.e., PCG with **M**^−1^ = **I**) and inverse from Cholesky (or QR) decomposition of **A**^−1^. Rather than direct inversion, a symmetric positive-definite matrix like **A**^−1^ can be inverted from its Cholesky decomposition. Matrix **A**^−1^ can be decomposed into a product of **A**^−1^ = **U**′**U**, where **U** is an upper triangle matrix with non-zero diagonal elements. Then, **A** is calculated from **U**, (**A** = **U**^−1^(**U**^−1^)^′^). Function chol2inv from R package “Matrix” (Bates and Maechler, 2019) inverts a matrix from its Cholesky decomposition. Also, R function chol computes the Cholesky decomposition of a matrix. Computational cost for direct inversion of a matrix is *n*^3^, where *n* is the size of the matrix. Computational cost of Cholesky decomposition of a matrix (calculating **U**) is approximately (2*n*^3^+3*n*^2^)/6 (van de Geijn, 2011), and inverting a triangle matrix has a computational cost of *n*^2^. Although the computational cost of multiplying two *n*×*n* matrices is *n*^3^, considering one matrix being lower triangle and the other being upper triangle, the matrix multiplication cost can be reduced to $\overset{n}{\underset{i=1}{\sum }}i^{2}$. Using sparse matrix libraries (e.g., SuiteSparse, BLAS, and LAPACK) for sparse matrices, the computational costs would be less than those mentioned above.

Henderson (1976) presented a simple method for calculating **A**^−1^ by deriving its decomposition indirectly (**A**^−1^ = (**T**^−1^)^′^**D**^−1^**T**^−1^), where **D** is as defined in Thompson's Method, and **T**^−1^ = **I**−**J**, where **J** is a lower triangle parent incidence matrix with coefficients 0.5 and 0. Unfortunately, ${\text{U}}_{2}={\left({\text{T}}^{-1}\right)}^{\prime }{\text{D}}^{-\frac{1}{2}}$ cannot serve as an indirect Cholesky decomposition of **A**^−1^, because with **U**_1_ being the direct Cholesky decomposition of **A**^−1^, ${\text{A}}^{-1}={\text{U}}_{1}^{\prime }{\text{U}}_{1}={\text{U}}_{2}{\text{U}}_{2}^{\prime }$, **U**_1_≠**U**_2_, and ${\text{U}}_{1}\neq {\text{U}}_{2}^{\prime }$.

Another method is forward and backward substitution. This method consists of a backward substitution followed by a forward substitution, per column of **A**. In forward substitution, the equation system **Kc** = **b** is solved, where **b** is a vector of **0** with the *i*th element (corresponding to the *i*th column of **A**) set to 1, and **K** is a lower triangle matrix with non-zero diagonal elements, from the Cholesky decomposition of **A**^−1^ (*i.e*., **A**^−1^ = **KK**′). Then, in the backward substitution, the equation system **K**′**x** = **c** is solved, where **x** is the *i*th column of **A**. This method is coded in R function fbs (Data Availability Statement), and it requires matrix **A**^−1^ as input. We tested this method on PED1, PED2, and PED3 (results not shown). The results were good in terms of accuracy (RMSE), but the performance was poor in terms of computational time.

Another algorithm is based on the decomposition of **A**^−1^ to **T**^′−1^**D**^−1^**T**^−1^ (Colleau, 2002). This algorithm is used to create the product of **A** with a vector **x**, in linear time proportional to the number of animals. It can also be used to compute products of partitions of **A** multiplied by a vector (Misztal et al., 2009). For example, in genetic evaluations including pedigree and genomic information, the partition of **A** for genotyped animals is created by repeatedly applying the algorithm of Colleau (2002), where the multiplying vector is a vector of zeros containing a single 1 (Misztal et al., 2009), similar to vector **b** used in Solving **A**^−1^ for Columns of **A**.

### 6.2. Calculating Numerator Relationships From Inbreeding Coefficients

Van Vleck (2007) introduced a method for computing numerator relationships between any pair of animals. This method is based on the fact that the relationship between two individuals is twice the inbreeding coefficient of their progeny. As many pairs of animals do not have progeny (including animals from the same sex), this method involves introducing dummy progeny to pedigree for pairs of animals, for which calculating relationship coefficients is of interest. We did not test the performance of this method. It is expected to be very efficient for a small subset of pedigree, but not for a whole large pedigree, mainly due to the very many dummy progeny to be appended to the pedigree.

### 6.3. Optimizations for Less Memory Usage

Memory usage and the required disk space for saving matrix **A** to an external file can be reduced by calculating **A** in a tabular-sparse (non-zero elements and their row and column indices) rather than a matrix format, which is implemented in function tabA from R package “ggroups” (Nilforooshan and Saavedra-Jiménez, 2020). Although this method is memory efficient, it is recommended when the memory is a bottleneck. It reduces the memory usage at the cost of increased computational time. However, nowadays, memory is a relatively cheap component compared to other hardware components. In a previous study (Nilforooshan and Saavedra-Jiménez, 2020), calculating **A** for a pedigree of 3,000 animals took 13 min 56 s vs. 2 s, time for tabA vs. buildA (Tabular Method). Where matrix **A** needs to be saved in tabular-sparse format (takes less disk space), it can be built using function buildA or function tabularA (Data Availability Statement), converted to tabular-sparse format using function mat2tab from R package “ggroups” (Nilforooshan and Saavedra-Jiménez, 2020) and written to an external file. Writing the file in a binary format can further reduce the disk space usage.

In order to create and store matrix **A** compactly, but in a dense format, it can be created and stored in a lower triangle packed storage format (https://en.wikipedia.org/wiki/Packed_storage_matrix). That means, the lower triangle of matrix **A** is created and stored row-wise in a vector, where element *A*_*ij*_(*i*>*j*) is located at the position (*i*(*i*−1)/2)+*j* of the vector. This method is mainly adopted for storing symmetric dense matrices in an external file. For writing symmetric semi-sparse matrices like **A** in an external file, it has to be dense enough (> 1/3 of the upper/lower triangle elements) to choose this format over the tabular-sparse format. This method is coded in R function getAvec (Data Availability Statement). Calculating **A** using this method took less memory. However, the computational time increased to 24 min 6 s for PED4 on an “r5.4xlarge” machine with 128 Gb of RAM. Although the memory usage was reduced, 128 GB of RAM was still not enough and the procedure made use of a swap space of 12 Gb, which was partly responsible for the increased computational time. This method was tested for PED1, PED2, and PED3 on a “t2.medium” cloud machine with 2 cores and 4 Gb of RAM, and the calculation of **A** took 17 s for each pedigree. Repeating the same process on a “t2.small” machine with 1 core and 2 Gb of RAM, using a swap space for compensating the shortage of RAM, increased the elapsed time to 1 min 30 s, 1 min 5 s, and 1 min 6 s for PED1, PED2, and PED3, respectively. Therefore, using swap space for computations should be avoided as it noticeably slows down the procedure, unless only marginal excess memory above the provided RAM is required.

There might be situations, where the whole **A** is not needed, but a block of it. These situations may bring opportunities to reduce the computational complexity and memory demand. Supplementary Table 1 provides examples for Thompson's Method and Henderson's Method to calculate different blocks of **A**.

## 7. Concluding Remarks

Modifying the way of coding one of the oldest methods of calculating **A** (Tabular Method), this matrix was calculated in a shorter time than the time spent for calculating **A**^−1^. Computational time and memory usage are the main bottlenecks for calculating **A**. There is a trade-off between these two. Fast computation can be achieved by providing sufficient memory, and memory bottleneck can be lifted if computational time is not a hard limit. Creating and storing matrix **A** in a dense vector (packed storage) or in the tabular-sparse format can reduce the memory requirement, but the computational time is compromised.

Opportunities to speed up the calculation **A** by parallel processing were limited to a few methods. For most of the methods, calculations are iterative and not independent from each other (progeny following parents and generation after generation). Explicit parallelism (parallelism of a concurrent computation) does not seem to be an efficient option, because calculation of **A** involves many small and simple computations rather than a few large and complicated computations, where explicit parallel processing might be helpful (e.g., direct inversion of **A**^−1^). Therefore, parallelism overhead (time required to coordinate parallel tasks among computational nodes) is expected to be higher than the gain from parallelism.

The choice of programming language influences the computational time. However, comparing methods, time rankings, and accuracy matter the most. Programming languages with slow loops penalize iterative procedures. There are various optimization techniques available that can be applied to different conditions and limitations. Some examples are as follows: (1) Iteratively creating matrices **T** and **L**, we could not take advantage of libraries for sparse matrices. Those libraries were used to create **A** after **T** or **L** became available. In the shortage of memory, those matrices can be built in tabular-sparse format (e.g., three growing vectors of matrix elements and their row/column indices, then making a sparse matrix at once given those vectors). (2) We used vectorized operations to calculate multiple elements in a matrix row simultaneously. In species with multiple progeny per mating, this can be extended to multiple rows for full-sib progeny. In the same manner, multiple elements of vector **d** can be calculated simultaneously. (3) To reduce memory demand and computational complexity, small elements (e.g., <1e–5) can be set to zero on the fly.

Finally, in the absence of pedigree corrections for animals that are already in the pedigree, matrix **A** can be updated for new animals added to the pedigree. Therefore, calculations are done only once for each individual. Evidently, for updating **A** for each new individual, only coefficients corresponding to its parents (if known) are to be retrieved. Similarly, for pedigree corrections, **A** is corrected from where the pedigree is corrected. If relationship coefficients are no longer needed for old/culled animals, only the partition of **A** for animals chosen to be the parents to the next generation can be kept and saved in an external file. Other relationship coefficients can be archived in a separate file. Besides saving disk space, computational time and memory usage for the upcoming update of the **A** matrix are reduced.

## Data Availability Statement

The data, scripts and functions that support the findings of this study are openly available in the figshare repository https://doi.org/10.6084/m9.figshare.13497939.

## Author Contributions

MN initiated the idea of the study, wrote the programs, run the analyses, drafted the manuscript, and revised it. DG helped MN with more ideas to test and revising the manuscript. BH was the project leader and he was involved in the revision of the manuscript. All authors contributed to the article and approved the submitted version.

## Conflict of Interest

MN and BH are employed at the company Livestock Improvement Corporation, Hamilton, New Zealand. The remaining author declares that the research was conducted in the absence of any commercial or financial relationships that could be construed as a potential conflict of interest.

## Publisher's Note

All claims expressed in this article are solely those of the authors and do not necessarily represent those of their affiliated organizations, or those of the publisher, the editors and the reviewers. Any product that may be evaluated in this article, or claim that may be made by its manufacturer, is not guaranteed or endorsed by the publisher.

## Appendix

The R5 family instances of Amazon elastic compute cloud feature either the first- or second-generation Intel Xeon Platinum 8000 series processor with a sustained all core Turbo CPU clock speed of up to 3.1 GHz (https://aws.amazon.com/ec2/instance-types/r5/).

The C5 family instances of Amazon elastic compute cloud feature custom second-generation Intel Xeon Scalable Processors with a sustained all-core turbo frequency of 3.6 GHz and maximum single core turbo frequency of 3.9 GHz (https://aws.amazon.com/ec2/instance-types/c5/).

The T2 family instances of Amazon elastic compute cloud are low-cost general-purpose machines backed by Intel Xeon processors that provide a baseline level of CPU performance with the ability to burst to full core (3.3 GHz) performance (https://aws.amazon.com/ec2/instance-types/t2/).
