# Supplementary material for: Alternative Ways of Computing the Numerator Relationship Matrix
Source: Front Genet. 2021 Jul 28;12:655638. doi: 10.3389/fgene.2021.655638 (PMC8356081; doi:10.3389/fgene.2021.655638)
Supplement: Supplementary file 1 [file Table_1.pdf]

Supplementary Material

Table S1. Matrices to build and multiplications for the calculation of different blocks of **A** (the numerator relationship matrix), where $i < j < k < l$ and n is the size of the matrix.

Method	Block	To build	Multiplications
$\mathbf{A} = \mathbf{L}\mathbf{L}'$	$\mathbf{A}_{[i:j,i:j]}$	$\mathbf{L}_{[1:j,1:j]}$	$\mathbf{L}_{[i:j,1:j]}\mathbf{L}'_{[i:j,1:j]}$
	$\mathbf{A}_{[i:j,i:k]}$	$\mathbf{L}_{[1:k,1:j]}$	$\mathbf{L}_{[i:j,1:j]}\mathbf{L}'_{[i:k,1:j]}$
	$\mathbf{A}_{[i:j,i:n]}$	$\mathbf{L}_{[1:n,1:j]}$	$\mathbf{L}_{[i:j,1:j]}\mathbf{L}'_{[i:n,1:j]}$
	$\mathbf{A}_{[i:j,k:l]}$	$\mathbf{L}_{[1:l,1:j]}$	$\mathbf{L}_{[i:j,1:j]}\mathbf{L}'_{[k:l,1:j]}$
	$\mathbf{A}_{[i:j,k:n]}$	$\mathbf{L}_{[1:n,1:j]}$	$\mathbf{L}_{[i:j,1:j]}\mathbf{L}'_{[k:n,1:j]}$
	$\mathbf{A}_{[i:j,1:n]}$	$\mathbf{L}_{[1:n,1:j]}$	$\mathbf{L}_{[i:j,1:j]}\mathbf{L}'_{[1:n,1:j]}$
$\mathbf{A} = \mathbf{T}\mathbf{D}\mathbf{T}'$	$\mathbf{A}_{[i:j,i:j]}$	$\mathbf{T}_{[1:j,1:j]}$, $\mathbf{D}_{[1:j,1:j]}$	$\mathbf{T}_{[i:j,1:j]}\mathbf{D}_{[1:j,1:j]}\mathbf{T}'_{[i:j,1:j]}$
	$\mathbf{A}_{[i:j,i:k]}$	$\mathbf{T}_{[1:k,1:j]}$, $\mathbf{D}_{[1:j,1:j]}$	$\mathbf{T}_{[i:j,1:j]}\mathbf{D}_{[1:j,1:j]}\mathbf{T}'_{[i:k,1:j]}$
	$\mathbf{A}_{[i:j,i:n]}$	$\mathbf{T}_{[1:n,1:j]}$, $\mathbf{D}_{[1:j,1:j]}$	$\mathbf{T}_{[i:j,1:j]}\mathbf{D}_{[1:j,1:j]}\mathbf{T}'_{[i:n,1:j]}$
	$\mathbf{A}_{[i:j,k:l]}$	$\mathbf{T}_{[1:l,1:j]}$, $\mathbf{D}_{[1:j,1:j]}$	$\mathbf{T}_{[i:j,1:j]}\mathbf{D}_{[1:j,1:j]}\mathbf{T}'_{[k:l,1:j]}$
	$\mathbf{A}_{[i:j,k:n]}$	$\mathbf{T}_{[1:n,1:j]}$, $\mathbf{D}_{[1:j,1:j]}$	$\mathbf{T}_{[i:j,1:j]}\mathbf{D}_{[1:j,1:j]}\mathbf{T}'_{[k:n,1:j]}$
	$\mathbf{A}_{[i:j,1:n]}$	$\mathbf{T}_{[1:n,1:j]}$, $\mathbf{D}_{[1:j,1:j]}$	$\mathbf{T}_{[i:j,1:j]}\mathbf{D}_{[1:j,1:j]}\mathbf{T}'_{[1:n,1:j]}$
